# Effects of β_1_-adrenergic receptor blockade on the cerebral microcirculation in the normal state and during global brain ischemia/reperfusion injury in rabbits

**DOI:** 10.1186/s40360-020-0394-7

**Published:** 2020-02-21

**Authors:** Nobumasa Asano, Sohei Hishiyama, Tadahiko Ishiyama, Masakazu Kotoda, Takashi Matsukawa

**Affiliations:** 1 Department of Anesthesiology, Faculty of Medicine, University of Yamanashi, 1110 Shimokato, Chuo, Yamanashi, 409-3898 Japan; 2 F.M. Kirby Neurobiology Center, Boston Children’s Hospital, Harvard Medical School, 3 Blackfan Circle, Boston, MA 02115 USA

**Keywords:** β_1_-adrenergic receptor blockade, Ischemia/reperfusion injury, Cerebral microvasculature, Vasodilation

## Abstract

**Background:**

Although recent studies using experimental models of ischemic brain injury indicate that systemically-administered β_1_-blockers have potential protective effects on the cerebrovascular system, the precise mechanisms remain unclear. In addition to their cardiovascular effects, water-soluble β_1_-blockers can pass the blood–brain barrier and may exert their vascular action on cerebral microvessels. The aim of this study was to investigate the direct effects of β_1_-blockade on the cerebral microvasculature both in the normal state and ischemia/reperfusion state using the cranial window method.

**Methods:**

The closed cranial window method was used to visualize the cerebral microcirculation and changes in the pial arteriole diameter in adult male rabbits. In the first experiment, various concentrations of the selective β_1_-blocker landiolol were administered into the cranial window to evaluate the dose-response. In the second experiment, the effect of β_1_-blockade on the brain during ischemic/reperfusion injury was investigated. Global brain ischemia/reperfusion was induced by clamping the brachiocephalic, left common carotid, and left subclavian arteries for 15 min. Either landiolol or artificial cerebrospinal fluid was infused 5 min after initiation of ischemia through 120 min after reperfusion. Pial arteriole diameter and hemodynamic and physiological parameters were recorded before ischemia, during ischemia, and 5, 10, 20, 40, 60, 80, 100, and 120 min after reperfusion.

**Results:**

In the first experiment, topical administration of landiolol at higher concentrations produced slight pial arteriole dilation (10^− 8^ mol/L: 4.3 ± 3.4%, 10^− 6^ mol/L: 8.0 ± 5.8%, 10^− 4^ mol/L: 7.3 ± 4.0%). In the second experiment, the topical administration of landiolol significantly dilated the pial arteriole diameters during ischemia/reperfusion injury (ischemia: 30.6 ± 38.6%, 5 min: 47.3 ± 42.2%, 10 min: 47.8 ± 34.2%, 20 min: 38.0 ± 39.0%). There were no statistical differences in hemodynamic and physiological parameters between the landiolol and control groups.

**Conclusions:**

The blockade of β_1_-adrenergic receptors induced significant vasodilation of pial arterioles during ischemia/reperfusion injury. By contrast, only a slight dilation of the arterioles was observed in the normal state, indicating that ischemic cerebral microvessels are more susceptible to the vasodilatory effect induced by selective blockade of β_1_-adrenergic receptors than normal microvessels.

## Background

Ischemic brain injury is a major cause of death and morbidity worldwide. Although numerous neuroprotective agents have been investigated, few of them have been shown to impact the clinical outcomes of ischemic brain injury [1]. Recent studies using experimental models of ischemic brain injury indicate that systemically-administered β_1_-blockers have potential protective effects on the cerebrovascular system [2, 3]. Although several possible mechanisms underlying the neuroprotective effects of β_1_-blockers, such as suppression of apoptosis or inflammatory responses, have been proposed, the precise mechanisms remain unveiled. In addition to their cardiovascular effects, water-soluble β_1_-blockers can pass the blood–brain barrier and may exert its vascular action on the cerebral microvasculature [4]. Local blockade of β_1_-adrenergic receptors in the brain may lead to the dilation of cerebral microvasculature via suppression of norepinephrine release [5] and enhancement of endothelium-derived hyperpolarization [6, 7]. Despite the potential clinical and pharmacological impact of β_1_-blockade on the outcomes of ischemic brain injuries, its direct local effects on cerebral microvasculature have not been investigated. The aim of this study was to investigate these effects both in the normal state and ischemia/reperfusion state using the cranial window method in rabbits.

## Methods

The experiments were conducted in accordance with the National Institutes of Health guidelines for the care and use of laboratory animals. The experimental protocol was reviewed and approved by the University of Yamanashi Animal Care Committee. All animals were euthanized with a pentobarbital sodium overdose after experiments.

### Experimental preparation

Male Kbs:JW rabbits (Kitayama Labes, Nagano, Japan) weighing 2.8–3.8 kg were used in this study. General anesthesia was induced and maintained using intravenous pentobarbital sodium via the ear vein (20 mg/kg for induction and 5 mg/kg/h for maintenance). Tracheostomy was performed and each animal’s lungs were placed under mechanical ventilation. The respiratory rate and tidal volume were adjusted to maintain the arterial CO_2_ tension (PaCO_2_) between 35 and 45 mmHg. End-tidal CO_2_ (EtCO_2_) was monitored using a capnogram (Vamos, Dräger medical, Tokyo, Japan) during mechanical ventilation. Mean arterial blood pressure (MAP) and blood gas analysis were measured continuously using a catheter inserted in the femoral artery (either one). The rectal temperature was monitored and maintained at 39 ± 0.5 °C during the experiments using a heat pad. All experiments were conducted between 09:00 and 17:00 under normal room light and temperature (23 ± 2 °C).

### Cranial window installation

The closed cranial window method was used to visualize the pial microcirculation as described previously [8]. Animals were placed in the prone position under pentobarbital anesthesia. An 8-mm burr hole was made in the parietal bone using a dental micro drill, and the dura and arachnoid membranes were cauterized and cut to expose the pial vessels. A thin glass disk was attached over the burr hole using dental acrylic and bone wax so that the fluid volume under the glass disk was 0.5–0.7 mL. Two inlets and one outlet were created using thin polyethylene catheters. One inlet was connected to a bottle containing artificial cerebrospinal fluid (CSF) that contained the following: Na^+^, 151 mEq/L; K^+^, 3.5 mEq/L; Ca^2+^, 2.5 mEq/L; Mg^2+^, 1.3 mEq/L; HCO_3_^−^, 25 mEq/L; glucose, 65 mg/dL; and urea, 40 mg/dL (continuously bubbled with 5% CO_2_ in air). The artificial CSF was suffused at a rate of 0.1 mL/min. The drug solutions to be tested were administered via the other inlet catheter. The exit of the outlet catheter was placed 5–6 cm above the window to maintain the normal intracranial pressure. The pial microvessels were visualized using a digital video analyzer (VH Analyzer VH-H1A5, Keyence, Osaka, Japan) and video capture unit (VH-E500, Keyence, Osaka, Japan) and the diameter of the arterioles was measured using still images.

### Ischemic brain injury

**Fig. 1:**
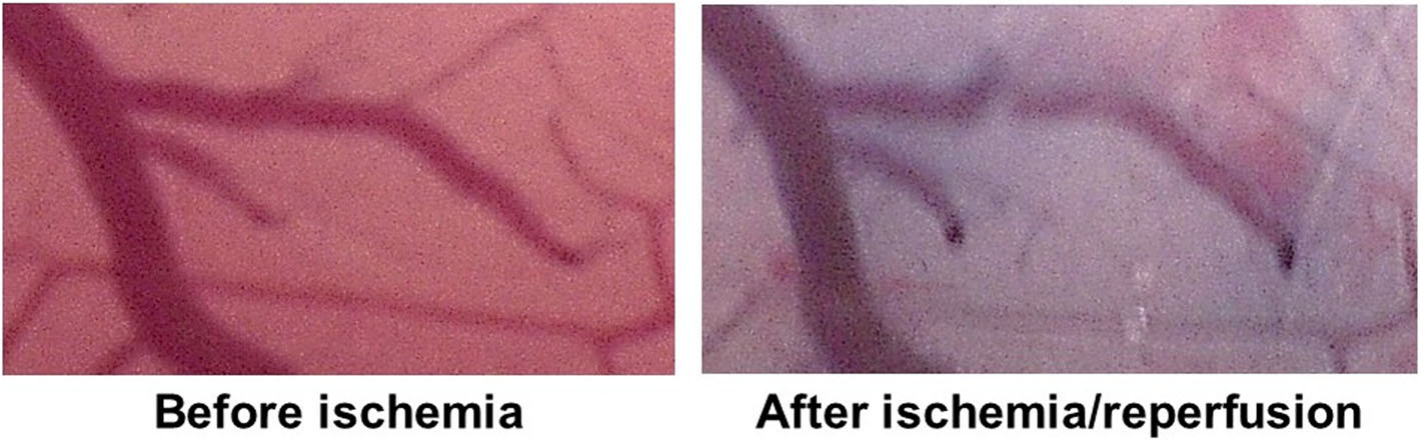
Blood–brain barrier disruption after ischemic/reperfusion Representative images of pial microvessels before and after ischemia/reperfusion. Leakage of Evans blue dye indicates the increased vascular permeability after ischemic/reperfusion

Global brain ischemia/reperfusion injury was induced by clamping the main branches of the aorta (brachiocephalic, left common carotid, and left subclavian arteries) for 15 min as described previously [9]. The cerebral blood flow (CBF) was measured in another set of three rabbits to verify brain ischemia/reperfusion in this model. A 1-mm burr hole was made in the parietal bone using a micro drill, and a laser Doppler flowmeter (FLO-C1; Omegaflo, Tokyo, Japan) was inserted perpendicular to the bone surface. The rabbits were subjected to artery clamping for 15-min after Doppler flowmeter installation. The CBF decreased by 89.3 ± 9.2% (mean ± standard deviation) after clamping, and recovered after unclamping, confirming that three-vessel clamping induces brain severe brain ischemia and reperfusion. Moreover, vascular permeability after ischemia/reperfusion was assessed using Evans blue extravasation [10]. Briefly, 2% Evans blue 1 mL/kg (Sigma-Aldrich, St. Louis, MO, USA) in normal saline was administered intravenously, immediately after the ischemia/reperfusion. Figure 1 shows a representative picture of Evans blue extravasation, confirming the increase in vascular permeability caused by ischemia/reperfusion in this model.

### Experiment 1 (normal state)

The cranial window was superfused for 5 min (at 0.1 mL/min for the first 1 min and at 0.1 mL/min for the next 4 min) with each of six increasing concentrations (10^− 14^, 10^− 12^, 10^− 10^, 10^− 8^, 10^− 6^, and 10^− 4^ mol/L) of the selective β_1_-blocker landiolol (dissolved in artificial CSF, *n* = 10) (Ono Pharmaceutical, Osaka, Japan) after the baseline measurement of hemodynamic parameters and the pial arteriole diameter,. The diameter of the pial arterioles, MAP, heart rate (HR), and physiological parameters were recorded 5 min after application of each concentration. The window was flushed with artificial CSF for 30 min before testing the next concentration.

### Experiment 2 (ischemia/reperfusion injury)

Prior to the experiment, the animals were randomly assigned to two experimental groups: the control and landiolol groups (*n* = 4 each). Artificial CSF was infused into the cranial windows in the control group. Landiolol at 10^− 6^ mol/L was infused 5 min after the initiation of global brain ischemia through 120 min after reperfusion in the landiolol group. The diameter of the pial arterioles and hemodynamic and physiological parameters were recorded at the following time-points: right before the onset of global brain ischemia (baseline), 10 min after onset (ischemia), and 5, 10, 20, 40, 60, 80, 100, and 120 min after reperfusion.

### Statistical analysis

Statistical analysis was performed using Stat Flex version 6.0 (Artec, Osaka, Japan). All values are presented as the mean ± standard deviation. One-way or two-way analysis of variance for repeated measures followed by the Dunnett test was used to analyze the pial arteriole diameter and other variables. A *p*-value of < 0.05 was considered statistically significant. Power analysis indicated that the sample size of 4 animals per group was sufficient to achieve 80% power, with an α level of 0.05 to detect a mean difference of 10% in arteriole diameter.

## Results

### Effect of topical administration of landiolol in the normal state

**Fig. 2:**
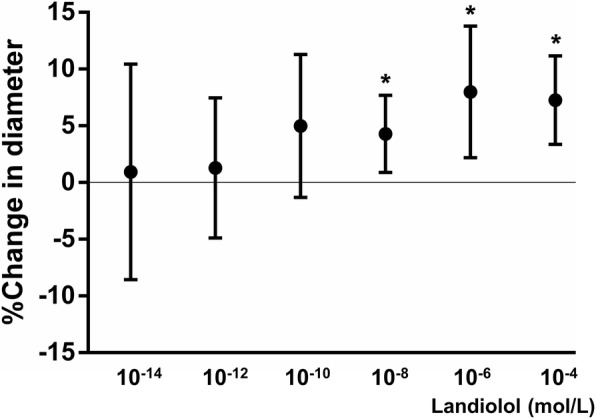
Effect of topical administration of landiolol in the normal state The topical administration of landiolol at higher concentrations produced a slight pial arteriole dilation (10^− 8^: 4.29 ± 3.36%, 10^− 6^: 7.99 ± 5.82%, 10^− 4^: 7.26 ± 3.99%, *: *p* < 0.05)

As shown in Fig. 2 and Table 1, topical administration of landiolol at lower concentrations (10^− 14^–10^− 10^ mol/L) did not produce significant changes in the pial arteriole diameter. However, when administered at higher concentrations (10^− 8^–10^− 4^ mol/L), landiolol produced a slight pial arteriole dilation (10^− 8^ mol/L: 4.3 ± 3.4%, 10^− 6^ mol/L: 8.0 ± 5.8%, 10^− 4^ mol/L: 7.3 ± 4.0%). The MAP, HR, arterial pH, base excess (BE), PaCO_2_, arterial oxygen tension (PaO_2_), as well as the plasma Na^+^, K^+^, and glucose concentrations did not change significantly during the experiment.

**Table 1:** Hemodynamic and laboratory data in Experiment 1

	MAP	HR	pH	BE	PaCO2	PaO2	Na	K	Glucose
	(mmHg)	(beats/min)			(mmHg)	(mmHg)	(mEq/L)	(mEq/L)	(mg/dL)
10^−14^	94 ± 12	233 ± 62	7.39 ± 0.04	−1.5 ± 3.1	38.5 ± 2.3	351 ± 84	140 ± 7	3.5 ± 0.8	149 ± 17
10^− 12^	97 ± 9	212 ± 49	7.38 ± 0.04	−0.3 ± 1.5	42.3 ± 3.1	433 ± 41	139 ± 5	3.5 ± 0.6	129 ± 11
10^−10^	95 ± 4	95 ± 4	7.38 ± 0.05	− 0.5 ± 4.4	42.3 ± 0.8	367 ± 130	145 ± 3	3.3 ± 0.6	144 ± 15
10^−8^	101 ± 7	101 ± 7	7.39 ± 0.03	0.0 ± 1.8	41.9 ± 1.6	423 ± 43	140 ± 4	3.5 ± 0.3	125 ± 11
10^− 6^	97 ± 16	97 ± 16	7.42 ± 0.05	1.8 ± 1.9	40.0 ± 2.8	429 ± 47	140 ± 3	3.2 ± 0.3	128 ± 15
10^−4^	96 ± 8	96 ± 8	7.41 ± 0.09	1.0 ± 5.0	40.0 ± 3.5	459 ± 84	142 ± 4	3.1 ± 0.1	154 ± 65

Values are expressed as mean ± SD

There were no statistical differences among the concentrations

*MAP* mean arterial pressure; *HR* heart rate; *BE* base excess

### Effect of topical administration of landiolol during ischemic/reperfusion injury

The results of Experiment 1 indicated that landiolol at the concentration of 10^− 6^ mol/L produces a peak vasodilatory effect on cerebral pial arterioles. On the basis of this dose-ranging experiment, we selected 10^− 6^ mol/L as the concentration of landiolol for Experiment 2.

**Fig. 3:**
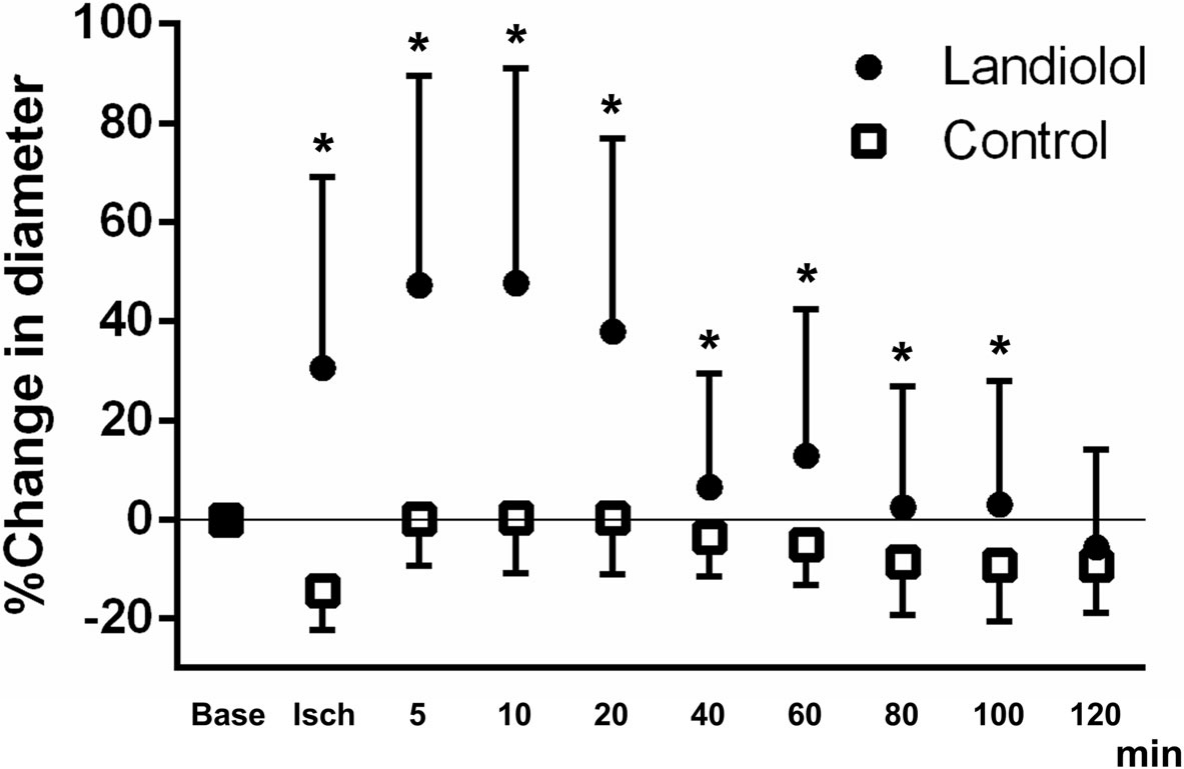
Effect of topical administration of landiolol during ischemic/reperfusion injury The topical administration of landiolol significantly dilated the pial arterioles during ischemia/reperfurion injury [ischemia (Isch): 30.6 ± 38.6%, 5 min: 47.3 ± 42.2%, 10 min: 47.8 ± 34.2%, 20 min: 38.0 ± 39.0%, 40 min: 6.6 ± 23.0%, 60 min: 12.8 ± 29.7%, 80 min: 2.5 ± 24.3%, 100 min: 3.1 ± 24.9%, *: *p* < 0.05 compared with control]. The vasodilatory effect of landiolol reaches a peak 5 to 10 min after injection, and pial arteriole diameter then gradually recovers to baseline (Base) level over 120 min. In the control group, the pial arterioles significantly constricted during global brain ischemia. The arteriole diameter recovers to baseline after unclamping, and then gradually decreases over 120 min

As shown in Table 2, the MAP significantly increased after clamping the brachiocephalic, left common carotid, and left subclavian arteries both in the control and landiolol groups. In contrast, the HR remained largely unchanged in both groups. After unclamping, the MAP, HR, and BE decreased, while plasma glucose increased significantly. There were no significant differences in hemodynamic and physiological variables between groups. As shown in Fig. 3 and Table 2, topical administration of landiolol significantly dilated the pial arterioles during ischemia/reperfurion injury (ischemia: 30.6 ± 38.6%, 5 min: 47.3 ± 42.2%, 10 min: 47.8 ± 34.2%, 20 min: 38.0 ± 39.0%, 40 min: 6.6 ± 23.0%, 60 min: 12.8 ± 29.7%, 80 min: 2.5 ± 24.3%, 100 min: 3.1 ± 24.9%). The vasodilatory effect of landiolol reached a peak 5 to 10 min after injection, and the pial arteriole diameter then gradually recovered to the baseline level over 120 min. In the control group, pial arterioles significantly constricted during global brain ischemia. The arteriole diameter recovered to baseline after unclamping and then gradually decreased over 120 min.

**Table 2:** Hemodynamic and laboratory data in Experiment 2

	MAP	HR	pH	BE	PaCO2	PaO2	Na	K	Glucose
	(mmHg)	(beats/min)			(mmHg)	(mmHg)	(mEq/L)	(mEq/L)	(mg/dL)
Control group									
baseline	107 ± 15	306 ± 45	7.43 ± 0.05	− 0.7 ± 2.1	40.0 ± 5.6	179 ± 14	141 ± 2	3.3 ± 0.3	141 ± 18
ischemia	115 ± 35	303 ± 55	7.37 ± 0.08	−6.3 ± 4.2	35.2 ± 9.0	115 ± 60	140 ± 4	3.7 ± 0.5	272 ± 84
Unclamp 5 min	91 ± 44	289 ± 52	7.30 ± 0.08	−7.3 ± 3.1	41.2 ± 3.7	160 ± 35	140 ± 3	3.7 ± 0.2	272 ± 88
Unclamp 10 min	88 ± 36	301 ± 59	7.30 ± 0.09	−7 ± 2.6	41.8 ± 4.6	148 ± 28	139 ± 2	3.4 ± 0.2	273 ± 110
Unclamp 20 min	86 ± 34	297 ± 59	7.32 ± 0.06	−6.8 ± 2.3	41.5 ± 0.6	152 ± 26	138 ± 2	3.5 ± 0.4	268 ± 116
Unclamp 40 min	77 ± 29	289 ± 55	7.35 ± 0.06	− 5.1 ± 3.5	39.2 ± 2.0	160 ± 33	139 ± 4	3.6 ± 0.5	242 ± 111
Unclamp 60 min	74 ± 25	282 ± 49	7.37 ± 0.05	− 3.7 ± 2.3	38.7 ± 1.8	170 ± 22	139 ± 3	3.9 ± 0.5	214 ± 87
Unclamp 80 min	79 ± 29	281 ± 48	7.38 ± 0.05	−3.1 ± 3.1	38.0 ± 1.8	167 ± 37	140 ± 3	4.0 ± 0.6	196 ± 73
Unclamp 100 min	78 ± 29	284 ± 57	7.38 ± 0.06	−4.3 ± 3.2	36.5 ± 2.8	158 ± 29	140 ± 2	4.0 ± 0.6	176 ± 53
Unclamp 120 min	79 ± 33	294 ± 75	7.38 ± 0.07	−4.6 ± 3.1	36.1 ± 2.6	165 ± 36	140 ± 4	4.0 ± 0.5	167 ± 44
Landiolol group									
baseline	91 ± 27	290 ± 33	7.43 ± 0.05	1.0 ± 1.2	38.2 ± 4.3	162 ± 37	143 ± 3	3.1 ± 0.5	131 ± 36
ischemia	122 ± 35	322 ± 90	7.34 ± 0.02	− 8.0 ± 1.4	31.7 ± 4.9	140 ± 32	141 ± 5	3.8 ± 1.1	200 ± 73
Unclamp 5 min	90 ± 17	295 ± 51	7.22 ± 0.04	− 9.3 ± 3.0	44.3 ± 4.2	141 ± 36	141 ± 5	3.5 ± 0.5	203 ± 56
Unclamp 10 min	96 ± 9	298 ± 45	7.23 ± 0.05	− 9.5 ± 3.3	43.3 ± 4.7	143 ± 48	141 ± 3	3.4 ± 0.6	209 ± 65
Unclamp 20 min	88 ± 12	290 ± 58	7.26 ± 0.09	− 9.3 ± 3.2	39.5 ± 4.2	139 ± 35	141 ± 4	3.3 ± 0.6	205 ± 59
Unclamp 40 min	96 ± 11	294 ± 53	7.28 ± 0.10	− 7.5 ± 3.1	40.9 ± 6.2	128 ± 35	141 ± 4	3.4 ± 0.7	211 ± 89
Unclamp 60 min	84 ± 6	294 ± 53	7.30 ± 0.06	− 6.0 ± 4.0	41.2 ± 3.8	134 ± 50	140 ± 4	3.6 ± 1.0	201 ± 106
Unclamp 80 min	81 ± 13	295 ± 49	7.31 ± 0.08	− 5.5 ± 5.1	40.7 ± 3.5	164 ± 43	140 ± 3	3.5 ± 0.7	204 ± 133
Unclamp 100 min	75 ± 18	291 ± 49	7.32 ± 0.11	−4.8 ± 5.7	40.5 ± 3.4	139 ± 61	141 ± 3	3.6 ± 0.8	166 ± 66
Unclamp 120 min	73 ± 14	295 ± 55	7.34 ± 0.11	−4.5 ± 5.7	38.9 ± 4.0	123 ± 43	140 ± 4	3.6 ± 0.9	162 ± 64

Values are expressed as mean ± SD

MAP significantly increased after clamping the brachiocephalic artery, left common carotid artery, and left subclavian artery both in the control and landiolol groups. In contrast, HR remained largely unchanged in both groups. After unclamping, MAP, HR, and BE decreased. Plasma glucose increased significantly. There were no significant differences in the hemodynamic and physiological variables between groups

*MAP* mean arterial pressure; *HR* heart rate; *BE* base excess

## Discussion

In the present study, we first demonstrated that the local blockade of β_1_-adrenergic receptors leads to vasodilation of pial arterioles especially during ischemia/reperfusion injury.

Based on the structural design of the cranial window, we assumed that most of the drug solution infused into the window was drained from the outlet catheter and not absorbed into the systemic circulation. Even if all the solution was absorbed, the average infusion rate used in the present study was 3.3 μg/kg/min (10^− 4^ mM), which is considered equivalent to the adult human dose of 1 μg/kg/min, based on the body surface area [11]. The infusion rate is smaller than that used in clinical settings (1–125 μg/kg/min), especially for young healthy animals that have no cardiac dysfunction. Because systemic hemodynamic parameters were not affected by the topical administration of landiolol, it appears that landiolol did not affect the systemic condition, and the pial vasodilation observed in this study reflects the direct local effects of selective β_1_-blockade on cerebral microvessels.

There are two possible mechanisms underlying the local vasodilatory effects of β_1_-blocade observed in this study, i.e. suppression of norepinephrine release [5] and enhancement of endothelium-derived hyperpolarization [6, 7].

Peripheral norepinephrine release or sympathetic nerve stimulation constricts the pial arterioles [5, 12]. Arterioles are small-diameter blood vessels in the arterial side of the microcirculation that lead to the capillaries. They generally consist of three vascular layers: a single epithelial layer (intima), several smooth muscle layers (media), and an outermost connective tissue layer (adventitia) [13]. The adventitia includes nerve endings, which play an important role in sympathetic regulation of vascular constriction and dilation [13]. Microvessels with smaller diameter are more susceptible to catecholamine-induced vasoconstriction [12]. A recent study revealed that the blockade of β_1_-adrenergic receptors lowers norepinephrine release by inhibiting presynaptic β_1_-adrenergic receptors [14].

Another regulatory system that mediates vascular relaxation of the arterioles is endothelium-derived hyperpolarization [6, 7], a vasodilatory mechanism that is independent of the nitric oxide pathway [6], and is predominant in the regulation of vascular relaxation than the nitric oxide pathway especially in the microvessels [6]. Vascular endothelial cells contain intermediate conductance Ca^2+^-activated potassium channels (IK_Ca_) [6, 15]. It is reported that the activation of β_1_-adrenergic receptors suppresses endothelial IK_Ca_ hyperpolarization and consequent vascular relaxation [6].

Considering the above, it is reasonable to assume that the selective blockade of β_1_-adrenergic receptors causes vascular relaxation via the mechanisms described above, i.e. by lowering norepinephrine release and enhancing the endothelial IK_Ca_ hyperpolarization.

In the normal state, landiolol produced the peak vasodilatory effect at a concentration of 10^− 6^ mol/L. Based on the molecular weight of landiolol (546 g/mol), 10^− 6^ mol/L is equivalent to 0.546 μg/mL. According to an earlier study, the human plasma concentration of landiolol reaches approximately 1 μg/mL when the drug is administered intravenously at a rate of 40 μg/kg/min, the maximum dose for maintenance in clinical settings, for 1 h [16]. Hence, the concentration of 10^− 6^ mol/L (0.546 μg/mL) represents a clinically relevant and high enough concentration, while 10^− 4^ mol/L represents a very high concentration. Accordingly, it would be reasonable to attribute the peak vasodilatory effect observed with 10^− 6^ mol/L to the ceiling effect of this drug at least in the present experimental setting. In contrast, we found that topical application of 10^− 6^ mol/L landiolol produces a significant vasodilation of the pial arterioles during and following ischemia/reperfusion injury, indicating that ischemic microvessels are more susceptible to the vasodilatory effect of β_1_-adrenergic receptor blockade than normal microvessels. The increased vascular permeability of ischemic arterioles observed in the present study and earlier studies may explain this difference [17]. Thus, we assume that extraluminally-administered landiolol penetrates into the ischemic arterioles and reaches the inner layers, where it exerts its vasodilatory activities, causing a remarkable dilation of arterioles.

Under ischemic conditions, arteriolar constriction and hypoperfusion contribute to development of cerebral edema [18, 19]. Vasodilation of constricted arterioles after brain ischemia may provide sufficient oxygen and glucose supply, thus maintaining neural function. The neuroprotective effects of landiolol after brain ischemia reported in the literature may be partly attributable to its local vasodilatory activity, as previous studies support that an increase in CBF leads to neuroprotection in experimental models of ischemic brain injury [20, 21].

In agreement with the above, the results of this study indicate that the blockade of β_1_-adrenergic receptors in the brain increases CBF. CBF is determined by the ratio of cerebral perfusion pressure (CPP) to cerebral vascular resistance (CVR), while CPP is defined as the difference between the MAP and intracranial pressure (or central venous pressure, whichever is greater) [22]. It is reported that arterioles, but not capillaries, are detarminant factors for vascular resistance [23]. Thus, relaxation of the cerebral arterioles may result in decreased CVR, and subsequently, increased CBF as the laminar flow is proportional to the fourth power of vessel radius (Poiseuille’s law). This hypothesis is supported by a previous study reporting that CBF measured by laser Doppler flowmetry and pial vascular diameter change similarly [18].

This study has several limitations. First, although the increased vascular permeability of the ischemic microvessels appears to be the responsible mechanism for the increased susceptibility to the vasodilatory effects of landiolol, we could not exclude possible changes in β_1_ receptors caused by ischemia/reperfusion. Second, we focused on the direct pharmacological effect of β_1_-blockade on ischemic cerebral microvessels, and we therefore used topical administration instead of systemic administration to evaluate these effects on cerebral microvasculature independently from the systemic conditions and hemodynamic parameters. Therefore, our findings may not be directly applicable to clinical practice. Third, although we demonstrated the direct local vasodilatory effects of β_1_-blockade, we did not examine the neuroprotective effect of β_1_-blockers. The vasodilation of cerebral vessels and subsequent increase in CBF during ischemic brain injuries can be both neuroprotective and harmful [24]. Further exploratory studies are required to assess the effects of β_1_-blockade on neurons and neurological functions, as well as to determine the conditions in which vasodilation provides neuroprotection, in order to elucidate the neurological outcomes and mechanisms underlying the neuroprotective effects of β_1_-blockers reported in the literature.

## Conclusions

The blockade of β_1_-adrenergic receptors induced significant vasodilation of pial arterioles during ischemia/reperfusion injury. By contrast, only a slight dilation of the arterioles was observed in the normal state, indicating that ischemic cerebral microvessels are more susceptible to the vasodilatory effect of the selective blockade of β_1_-adrenergic receptors compared to normal microvessels.

## Data Availability

The datasets used and/or analyzed during the current study are available from the corresponding author on reasonable request.

## Abbreviations

BE: Base excess
IK_Ca_ Ca^2+^: Activated potassium channels
CBF: Cerebral blood flow
CPP: Cerebral perfusion pressure
CSF: Cerebrospinal fluid
CVR: Cerebral vascular resistance
EtCO_2_: End-tidal CO_2_
HR: Heart rate
MAP: Mean arterial blood pressure
PaCO_2_: Arterial carbon dioxide tension
PaO_2_: Arterial oxygen tension
